# Use of traditional cooking fuels and the risk of young adult cataract in rural Bangladesh: a hospital-based case-control study

**DOI:** 10.1186/1471-2415-11-16

**Published:** 2011-06-16

**Authors:** Joydhan Tanchangya, Alan F Geater

**Affiliations:** 1 Impact "Jibon Tari" Floating Hospital, Impact Foundation Bangladesh, 7th Floor, House 23, Road 113/A, Gulshan-2, Dhaka-1212, Bangladesh; 2 Epidemiology Unit, Faculty of Medicine, Prince of Songkla University, Hat Yai, Songkhla, 90112, Thailand

**Keywords:** Young adult cataract, risk factor, traditional cooking fuels, Bangladesh

## Abstract

**Background:**

This study aimed to investigate the independent relationship between the use of various traditional biomass cooking fuels and the occurrence of cataract in young adults in rural Bangladesh.

**Methods:**

A hospital-based age- and sex-matched case-control study incorporating two control groups was conducted. Cases were cataract patients aged 18 and 49 years diagnosed on the basis of any opacity of the crystalline lens or its capsule and visual acuity poorer than 6/18 on the Log Mar Visual Acuity Chart in either eye, or who had a pseudophakic lens as a result of cataract surgery within the previous 5 years. Non-eye-disease (NE) controls were selected from patients from ENT or Orthopaedics departments and non-cataract eye-disease (NC) controls from the Ophthalmology department. Data pertaining to history of exposure to various cooking fuels and to established risk factors for cataract were obtained by face-to-face interview and analyzed using conditional logistic regression.

**Results:**

Clean fuels were used by only 4% of subjects. A majority of males (64-80% depending on group) had never cooked, while the rest had used biomass cooking fuels, mainly wood/dry leaves, with only 6 having used rice straw and/or cow dung. All females of each group had used wood/dry leaves for cooking. Close to half had also used rice straw and/or cow dung. Among females, after controlling for family history of cataract and education and combining the two control groups, case status was shown to be significantly related to lifetime exposure to rice straw, fitted as a trend variable coded as never, ≤ median of all exposed, > median of all exposed (OR = 1.52, 95%CI 1.04-2.22), but not to lifetime exposure to wood/dry leaves. Case status among females showed an inverse association with ever use of cow dung as a cooking fuel (OR 0.43, 95%CI 0.22-0.81).

**Conclusions:**

In this population, where cooking is almost exclusively done using biomass fuels, cases of young adult cataract among females were more likely to have had an increased lifetime exposure to cooking with rice straw fuel and not to have cooked using cow dung fuel. There is a possibility that these apparent associations could have been the result of uncontrolled founding, for instance by wealth. The nature of the associations, therefore, needs to be further investigated.

## Background

A recent population-based survey in Bangladesh estimated 650,000 people were blind aged 30 years and older, around 80% due to cataract, and 130,000 new cases developed annually [1,2]. The prevalence of bilateral blindness was 2.9%, of severe visual impairment 1.2%-2.0% and of visual impairment 8.4% [3].

While the age-specific prevalence of blindness in Bangladesh, as in other countries, was found to increase with increasing age, there are some indications that the incidence of young adult cataract may be higher than expected compared with the age distribution of cataract patients in other countries. Data from the Impact "Jibon Tari" Floating Hospital in Bangladesh reveal that cataract operations account for 90% of all eye surgery, and that 14% of these patients are aged between 18 and 45 years. This contrasts with the 3.8% of cataract surgery patients between the ages of 20 and 49 in USA [4]. Even though 66% of adults in the Bangladesh are below 45, compared with only 47% in the USA [5], the large discrepancy in proportions of young adults among cataract surgery patients suggests a higher relative incidence of cataract among young adults in Bangladesh, and thus the possible existence of some specific risk factors for early onset of cataract in this population.

Age-specific blindness in Bangladesh was also found to be higher in women and the illiterate and disadvantaged people [1]. Most of those women were of low socioeconomic status and commonly used wood, leaves, cow dung and rice straw for household cooking and from a young age. Exposure to cheaper cooking fuels has been identified to be a risk factor in India and Nepal [6,7] and is very common in rural Bangladesh. These exposures might, therefore, be related to the high incidence of young adult cataract in Bangladesh. Such exposures are potentially avoidable and their identification as risk factors for young adult cataract in the Bangladeshi setting could indicate possible approaches for future policy-making to prevent blindness.

Previous studies of risk factors for cataract have considered cataract in the elderly. In our study, by contrast, we focused on a younger age group to determine if exposures to traditional biomass cooking fuels are actually risk factors among this population in the rural Bangladesh setting.

## Methods

### Study setting

The study was designed as a hospital-based matched case-control study with two types of control and conducted from May to October 2009 in Impact "Jibon Tari" Floating Hospital, Bangladesh. The hospital is located on a river and moves to disadvantaged areas of the country at 5- to 6-month intervals. It aims to provide health services, both clinical and surgical, to address the problems of disability in remote areas of the country. At the time of the study, the hospital was located in Barisal district, in the south-west of Bangladesh, a region criss-crossed by many rivers.

### Study population

Subjects were recruited from males and females aged between 18-49 years who came to visit Impact "Jibon Tari" Floating Hospital, Bangladesh, during the study period. Owing to the relative paucity of healthcare facilities above the primary level in rural areas, the potential catchment area for patients is wide, covering much of the district. Information on the location of the hospital is disseminated through the local media.

Although over 90% of households in rural Bangladesh have been reported to use traditional fuel of some kind [8], exposure to any one type while actually doing the cooking was expected to be much less. The required sample size for each of the case, non-eye-disease control and non-cataract eye-disease control groups, was therefore based on having a power of 80% to detect an odds ratio of at least 2 if between one third and one half of controls were exposed and the correlation coefficient for fuel use between cases and controls was 0.1 [9]. The sample size was calculated to be 153 per group.

### Sample selection

#### Case recruitment

Ophthalmologic patients aged between 18-49 years who came to visit the eye specialist in Impact "Jibon Tari" Floating Hospital and diagnosed as cataract by clinical and slit-lamp examination results showing opacity of the crystalline lens or its capsule and visual acuity poorer than 6/18 on the Log Mar Visual Acuity Chart in either eye, or having a pseudophakic lens as a result of cataract surgery within the previous 5 years, were recruited as cases in this study. Patients having congenital cataract, severe mental disorder or cataract secondary to serious eye disease, such as glaucoma, diabetes retinopathy or severe injury, were excluded.

#### Non-eye-disease (NE) controls

Non-eye-disease control subjects were selected from patients without any eye problem but attending the ear, nose and throat or orthopedics departments in the same hospital and 1:1 matched to cases on age (within same 5-year age range) and sex. Inclusion criteria were: aged between 18 and 49 years, having no history of prior cataract/eye surgery and no eye problem within last 3 months. Known cases of myopia were excluded. Potential subjects were recruited from among out-patients fulfilling the inclusion criteria on the same or following working day as the case to which they were matched.

#### Non-cataract eye-disease (NC) controls

Non-cataract eye-disease controls were selected from among patients attending in the same hospital with an eye problem other than cataract and matched 1:1 with cases on age (within same 5-year age range) and sex. Inclusion criteria were: aged between 18 and 49 years, having no history of prior cataract/eye surgery. Recruitment of potential subjects was made from the first patient subsequent to case attainment who fulfilled the inclusion criteria.

All eye examinations were carried out by an ophthalmologist using same LogMar Visual Acuity Chart and slit-lamp and in a similar manner for both cases and controls. Matching was done to allow control of the potential confounding effect arising from age and sex (both documented risk factors for cataract and likely to be associated with exposure to different types of cooking fuel). The ideal control group would have comprised subjects representative of the same catchment population as the cases but not having cataract. However, hospital-based controls were chosen for this study to reduce the potential bias that could arise from a possible lower willingness of community-based controls to respond to the interviewer. A single group of controls representative of non-case patients would have included a large proportion of patients with other eye diseases as the provision of health services for eye disease is a major component of the health care provision of the "Jibon Tari" Hospital. The two controls groups were therefore recruited to avoid potential problems that could arise from any similarity in exposure history between cases and patients with other eye disease.

### Data collection

Data were collected using a structured questionnaire through personal face-to-face interview following clinical examination. The questionnaire covered general socio-demographic characteristics, details of cooking history and exposure to various cooking fuels, and other previously documented risk factors for cataract.

Exposure to a cooking fuel in this study was defined as use of the fuel by the person doing the cooking. History of exposure was recorded on a matrix table comprising fuel types in rows and age (years 11 to 49) in columns. Recall of exposure earlier than age 11 was considered to be unreliable. Information was also obtained regarding the number of cooking sessions per day, hours spent cooking per session and number of days when cooking was done per week. These data were used to derive parameters of frequency, intensity, duration and cumulative lifetime exposure to each cooking fuel type.

Information on other documented or potential risk factors for cataract, including a history of hypertension and diabetes, was obtained by interview and not verified independently. Family history of cataract referred to parents, grandparents and siblings.

### Statistical analysis

Data were entered using EpiData version 3.1 [10] and transferred into R version 2.10.0 [11] for cleaning, exploration and analysis. The distributions of variables were explored and summarized within each outcome group using mean and standard deviation or frequency. Tabulation of independent variables was performed for matched pairs of case vs non-eye-disease control and for case vs eye-disease control. In addition, patterns of fuel use among males and females were tabulated.

Exposure variables showing any indication of differing within the matched pairs in either comparison (p < 0.2), in addition to selected parameters of cooking fuel use, were included in initial sex-specific conditional logistic regression models for matched pairs of case vs non-eye-disease control and of case vs non-cataract eye-disease control. These models allowed the independent associations between use of each fuel type and case status, controlling for confounding effects among the fuels and of other covariates, to be revealed. The models were refined by successive removal of variables showing no statistically significant contribution to the fit of either model, other than the selected cooking fuel variables, which were retained in the models irrespective of their statistical significance. Likelihood ratio test p-values ≤ 0.05 were considered as indicating statistical significance.

Associations between case status and alternative, ordinal, parameters of cooking fuel use were then explored among these fuels to identify any dose-response relationships, first using all subjects and subsequently in the subset of female subjects. These ordinal exposure variables comprised age at first exposure, frequency (times per week during the years used), intensity (hours per week during the years used), duration (years) and lifetime exposure (lifetime hours of use). Each variable except age at first exposure was cut into three levels: never used, used for less than or equal to the median among all users, and greater than the median for all users. Age at first exposure was cut into never exposed, older than or equal to the median age of all users, and less than the median age of all users. These variables were fitted first in categorical form and subsequently, if appropriate, in trend-across-category form.

To evaluate the effect of matching, additional analyses were performed using unconditional logistic regression modelling including the same variables as above.

### Ethical considerations

The study was approved by the Ethics Committee of the Faculty of Medicine, Prince of Songkla University, Thailand, and oral approval was granted after a detailed presentation of the research proposal within the management of Impact Foundation Bangladesh, the authority of the study hospital, before conducting the study. All potential participants in the study were informed that the study was aimed at identifying certain behaviours that might increase the risk of their developing ailments common in rural Bangladesh. Written informed consent was requested from all potential respondents before their participation. For participants who were willing but could not sign, a finger print was taken after explaining the research process. Only after the patient gave documented consent was the interview conducted. Computerized data did not indicate the identity of any patient.

## Results

None of the subjects selected for inclusion in the study refused to participate. One subject who had been selected as a NE control had insufficient time to be interviewed and was replaced by the next eligible subject. A total of 459 subjects, including 153 cataract cases aged between 18-49 years with an equal number of each control group matched on age and sex, were recruited and interviewed. There were slightly more females than males. The mean age of all participants was 41.8 (SD 6.3) years and 30 percent were aged 40 or less. In all groups, most of the females were housewives, the commonest occupation among males was farmer, and about 80 percent were classified as having low socioeconomic status as measured on the modified Kuppuswami scale using education, occupation and income of the family head. Around 70 to 75 percent of subjects were classified as being underweight. Cases had less commonly received secondary or tertiary education than controls, particularly non-eye-disease controls, and more commonly reported a history of current or past smoking and a greater occupational exposure to sunlight. Cases also more frequently reported a family history of cataract (Table 1). Very few subjects reported a history of diagnosis of hypertension (2%) or diabetes mellitus (1%).

**Table 1:** Distribution of socio-demographic characteristics among cases and controls.

Variables			Non-eye-disease controls		Non-cataract eye-disease controls	
	**Cases**					
	N (%)		N (%)	p-value*	N (%)	p-value*
Age in years (mean ± SD)	42.0 ± 6.1		41.8 ± 6.6		41.5 ± 6.1	
Age group (years)						
18 - 19	1 (0.7)		1 (0.7)		4 (2.6)	
20 - 29	7 (4.6)		7 (4.6)		2 (1.3)	
30 - 39	22 (14.4)		31 (20.3)		28 (18.3)	
40 - 49	123 (80.4)		113 (73.9)		119 (77.8)	
Sex						
Male	73 (47.7)		73 (47.7)		73 (47.7)	
Female	80 (52.3)		80 (52.3)		80 (52.3)	
Religion				0.35		0.07
Muslim	91 (59.5)		83 (54.2)		75 (49.0)	
Others	62 (40.5)		70 (45.8)		78 (51.0)	
Marital status				0.71		0.40
Single	7 (4.6)		6 (3.9)		5 (5.3)	
Married	139 (90.8)		137 (89.5)		144 (94.1)	
Divorce/widow	6 (3.9)		10 (6.5)		4 (2.6)	
Education				<0.001		0.08
No education	61 (39.9)		54 (35.3)		55 (35.9)	
Primary	73 (47.7)		54 (35.3)		65 (42.5)	
Secondary	19 (12.4)		45 (29.4)		33 (21.6)	
Occupation				0.56		0.75
Farmer	26 (17.0)		30 (19.6)		26 (17.0)	
Housewife	76 (49.7)		69 (45.1)		72 (47.1)	
Labour	11 (7.2)		10 (6.5)		9 (5.9)	
Business	12 (7.8)		14 (9.2)		13 (8.5)	
Teacher	3 (2.0)		3 (2.0)		7 (4.6)	
Others	25 (16.3)		27 (17.6)		26 (17.0)	
Household members				0.93		0.64
≤4	39 (25.5)		40 (26.1)		41 (26.6)	
5-7	93 (60.8)		94 (61.4)		86 (56.2)	
>7	21 (13.7)		19 (12.4)		26 (17.0)	
Household income (in previous year) taka/month				0.99		0.04
≤3000	50 (32.7)		50 (32.7)		31 (20.3)	
3001-7500	66 (43.1)		65 (42.5)		84 (54.9)	
>7500	37 (24.2)		38 (24.8)		38 (24.8)	
Socioeconomic status				0.33		0.64
Lower	127 (83.0)		120 (78.4)		124 (81.0)	
Middle	26 (17.0)		33 (21.6)		29 (19.0)	
Family history of cataract				<0.001		<0.001
Yes	54 (35.3)		26 (17.0)		29 (19.0)	
No	99 (64.7)		127 (83.0)		124 (81.0)	
Hx of working in sunlight				0.05		0.58
Yes	125 (81.7)		110 (71.9)		121 (79.1)	
No	28 (18.3)		43 (28.1)		32 (20.9)	
Sunlight exposure (years)				0.03		0.08
>18	70 (45.8)		51 (33.3)		54 (35.3)	
Ex. ≤18	55 (35.9)		59 (38.6)		67 (43.8)	
Not exposed	28 (18.3)		43 (28.1)		32 (20.9)	
Smoking status				0.08		0.001
Current or past	55 (35.9)		45 (29.4)		36 (23.5)	
Never smoked	98 (64.1)		108 (70.6)		117 (76.5)	
Current no.cigarettes/day				0.72		0.03
>10	25 (16.3)		22 (14.4)		12 (7.8)	
≤10	17 (11.1)		15 (9.8)		17 (11.1)	
None	111 (72.5)		116 (75.8)		124 (81.0)	

* Likelihood ratio p-values obtained from univariate conditional logistic regression models.

Over half of the NE controls (56.2%) were diagnosed as having various ear diseases followed by nasal, throat, and orthopedic diseases. Among NC controls, refractive error, corneal diseases and conjunctival diseases were most common (Table 2).

**Table 2:** Distribution of diagnosed diseases among controls.

Non-eye-disease controls		Non-cataract-eye-disease controls	
	
Diseases	N (%)	Diseases	N (%)
Ear diseases	86 (56.2)	Refractive error	47 (30.7)
Nasal diseases	15 (9.8)	Corneal diseases	42 (27.5)
Throat diseases	13 (8.5)	Conjunctival diseases	30 (19.6)
Orthopedic diseases	11 (7.2)	Lacrimal tract disease	8 (5.2)
Others	28 (18.3)	Others	26 (17.0)

Cooking history and exposure to various cooking fuels among subjects in each group are shown in Table 3. About two thirds of subjects in each group reported a history of cooking, either regularly or occasionally. All females in the study had cooked, almost all regularly, whereas only 36%, 34% and 27% of males respectively among cases, NE and NC controls had a history of cooking, with a majority (70% to 85%) cooking only occasionally. The commonest fuel used for cooking in all groups was wood and/or dry leaves, followed respectively by cow dung and rice straw. Further analysis of cooking fuel exposure was confined to these three groups of cooking fuels. Gas or kerosene was used by only a small number of subjects in any group, comprising less than 4% of the total. The proportion of cases using rice straw was higher than that of either of the controls, whereas the proportion of cases using cow dung was lower than that in each control group. The lifetime duration of cooking activities was somewhat higher among cases than either of the control groups. Exposure parameters explored included age at first exposure, frequency of exposure (times per week), intensity of exposure (hours per week during the years used), duration from first to most recent exposure in years (irrespective of the frequency of intensity of exposure), and total lifetime exposure (hours of actual exposure). However, these differentials were not statistically significant.

**Table 3:** Distribution of parameters of cooking history and cooking fuel use among cases and controls.

Variables	Cases N (%)	Non-eye-disease controls		Non-cataract eye-disease controls	
			
		N (%)	p-value*	N (%)	p-value*
**Cooking history**					
Ever cooked			0.18		0.59
Yes	108 (70.6)	100 (65.4)		105 (68.6)	
No	45 (29.4)	53 (34.6)		48 (31.4)	
Frequency			0.56		1.00
Regular	87 (80.6)	84 (84.0)		83 (79.0)	
Occasional	21 (19.4)	16 (16.0)		22 (21.0)	
Place			0.22		0.16
Living house	33 (30.6)	21 (21.0)		20 (19.0)	
Separate house	75 (69.4)	79 (79.0)		85 (81.0)	
Duration			0.10		0.003
≤26 yr	53 (49.1)	48 (48.0)		65 (61.9)	
>26 yr	55 (50.9)	52 (52.0)		40 (38.1)	
**Wood/dry leaves**					
Ever used			0.05		0.45
Used	106 (69.3)	95 (62.1)		102 (66.7)	
Never used	47 (30.7)	58 (37.9)		51 (33.3)	
Age at 1^st ^exposure			0.15		0.42
Never exposed	47 (30.7)	58 (37.9)		51 (33.3)	
Exposed at ≤15 years	56 (36.6)	45 (29.4)		60 (39.2)	
Exposed at <15 years	50 (32.7)	50 (32.7)		42 (27.5)	
Frequency of use			0.12		0.63
Never used	47 (30.7)	58 (37.9)		51 (33.3)	
Used≤14 times/week	98 (64.1)	85 (55.6)		90 (58.8)	
Used>14 times/week	8 (5.2)	10 (6.5)		12 (7.8)	
Intensity of use			0.09		0.44
Never used	47 (30.7)	58 (37.9)		51 (33.3)	
Used≤20 hr/week	64 (41.8)	50 (32.7)		56 (36.6)	
Used>20 hr/week	42 (27.5)	45(29.4)		46 (30.1)	
Duration of use			0.15		0.02
Never used	47 (30.7)	58 (37.9)		51 (33.3)	
Used ≤26yr	52 (34)	43 (28.1)		61 (39.9)	
Used >26yr	54 (35.3)	52 (34.0)		41 (26.8)	
Life time exposure			0.15		0.74
Never used	47 (30.7)	58 (37.9)		51 (33.3)	
Used≤25000 hr	55 (35.9)	44 (28.8)		52 (34.0)	
Used>25000 hr	51 (33.3)	51 (33.3)		50 (32.7)	
**Rice straw**					
Ever used			0.05		0.27
Used	29 (19.0)	18 (11.8)		22 (14.4)	
Never used	124 (81.0)	135 (88.2)		131 (85.6)	
Age at 1^st ^exposure			0.03		0.22
Never exposed	124 (81.0)	135 (88.2)		131 (85.6)	
Exposed at ≤15 years	11 (7.2)	11 (7.2)		13 (6.5)	
Exposed at <15 years	18 (11.8)	7 (4.6)		9 (7.8)	
Frequency of use			0.14		0.19
Never used	124 (81.0)	135 (88.2)		131 (85.6)	
Used≤14 times/week	25 (16.3)	18 (11.8)		21 (13.7)	
Used>14 times/week	4 (2.6)	0 (0.0)		1 (0.7)	
Intensity of use			0.15		0.28
Never used	124 (81)	135 (88.2)		131 (85.6)	
Used≤20 hr/week	16 (10.5)	10 (6.5)		12 (7.8)	
Used>20 hr/week	13 (8.5)	8 (5.2)		10 (6.5)	
Duration of use			0.07		0.42
Never used	124 (81)	135 (88.2)		131 (85.6)	
Used ≤26yr	12 (7.8)	10 (6.5)		11 (7.2)	
Used >26yr	17 (11.1)	8 (5.2)		11 (7.2)	
Life time exposure			0.15		0.55
Never used	124 (81)	135 (88.2)		131 (85.6)	
Used≤24000 hr	12 (7.8)	7 (4.6)		9 (5.9)	
Used>24000 hr	17 (11.1)	11 (7.2)		13 (8.5)	
**Cow dung**					
Ever used			0.20		0.08
Used	26 (17.0)	34 (22.2)		37 (24.2)	
Never used	127 (83.0)	119 (77.8)		116 (75.8)	
Age at 1^st ^exposure			0.45		0.13
Never exposed	127 (83.0)	119 (77.8)		116 (75.8)	
Exposed at ≤14 years	14 (9.2)	18 (11.8)		25 (16.3)	
Exposed at <14 years	12 (7.8)	16 (10.5)		12 (7.8)	
Frequency of use			0.28		0.16
Never used	127 (83.0)	119 (77.8)		116 (75.8)	
Used≤14 times/week	24 (15.7)	34 (22.2)		34 (22.2)	
Used>14 times/week	2 (1.3)	0 (0.0)		3 (2.0)	
Intensity of use			0.42		0.22
Never used	127 (83.0)	119 (77.8)		116 (75.8)	
Used≤20 hr/week	11 (7.2)	16 (10.5)		15 (9.8)	
Used>20 hr/week	15 (9.8)	18 (11.8)		22 (14.4)	
Duration of use			0.44		0.12
Never used	127 (83.0)	119 (77.8)		116 (75.8)	
Used ≤26yr	10 (6.5)	14 (9.2)		19 (12.4)	
Used >26yr	16 (10.5)	20 (13.1)		18 (11.8)	
Life time exposure			0.45		0.19
Never used	127 (83.0)	119 (77.8)		116 (75.8)	
Used≤31500 hr	12 (7.8)	16 10.5)		20 (13.1)	
Used>31500 hr	14 (9.2)	18 (11.8)		17 (11.1)	

* Likelihood ratio p-values obtained from univariate conditional logistic regression models.

Patterns of exposure to traditional fuels differed between females and males (Table 4). Only males reported no exposure to any of the three traditional fuel types, more commonly in the NE and NC controls (79% and 70% respectively) than in the cases (64%). All of the remaining males had used wood/dry leaves as cooking fuel, but only 6 of these had ever used rice straw or cow dung. By contrast, all females had used wood or dry leaves for cooking, with around half of the cases and NE controls and over half of NC controls using rice straw and/or cow dung in addition. The most striking differences among the groups was the higher percentage of cases (17.5%) than of either control (6% and 9%) who used rice straw as the only additional fuel, and the lower percentage of cases (16%) than of controls (27% and 29%) who used cow dung as the sole additional fuel.

**Table 4:** Patterns of exposure to ever use of biomass fuels among females and males.

Pattern code	Ever exposed				Case	Non-eye-disease control	Non-cataract eye-disease control
					
	WD/DL	RS	CD		N (%)	N (%)	N (%)
Females							
4	+	+	+		12 (15.0)	11 (13.7)	14 (17.5)
3	+	+	-		14 (17.5)	5 (6.3)	7 (8.8)
2	+	-	+		13 (16.3)	22 (27.5)	23 (28.7)
1	+	-	-		41 (51.2)	42 (52.5)	36 (45.0)
Males							
4	+	+	+		1 (1.4)	1 (1.4)	0
3	+	+	-		2 (2.7)	1 (1.4)	1 (1.4)
1	+	-	-		23 (31.5)	13 (17.8)	21 (28.8)
0	-	-	-		47 (64.4)	58 (79.4)	51 (69.9)

WD/DL = wood and/or dry leaves, RS = rice straw, CD = cow dung.

Comparison of socio-demographic variables among cases and controls within each of the exposure patterns revealed that only education differed significantly across the groups, with no education being more common among cases than controls in exposure patterns 0 and 4 (data not shown).

Because of the different combinations of cooking fuels used by males and females, conditional logistic regression modelling of ever use of the various cooking fuels was performed separately for males and females. For the male model, the 6 males who had cooked with rice straw and/or cow dung were excluded. Traditional cooking fuel types were fitted in binary form (ever used vs never used) as appropriate for males and females. Other variables initially included in the models were religion, educational level, income of family, treatment or diagnosis of hypertension, family history of cataract, sunlight exposure in the workplace and smoking status, as the p-value for the univariate association of each of these variables with case status in comparison with at least one of the control groups was < 0.2.

After refinement, the variables remaining in the model for females, in addition to ever use of the traditional cooking fuels, were education level of the subject and family history of cataract. The same variables, with the addition of smoking status, were retained in the model for males (Table 5). Religion, family income, history of hypertension, and workplace exposure to sunlight were not significant in the multivariate setting.

**Table 5:** Conditional logistic models for ever use of cooking fuels.

Cooking fuel	Numbers of patients			Cases vs non-eye-disease controls		Case vs non-cataract eye-disease patients		Case vs combined controls	
				
	CS	NE	NC	a-OR (95% CI)	p- value*	a-OR (95% CI)	p- value*	a-OR (95% CI)	p- value*
Females									
Family h/o cataract									
No	53	66	67	1 (ref)	0.03	1 (ref)	0.02	1 (ref)	0.007
Yes	27	14	13	2.14 (1.03, 4.43)		2.66 (1.15, 6.15)		2.31 (1.25, 4.26)	
Formal education									
At least secondary	6	17	13	1 (ref)	0.02	1 (ref)	0.35	1 (ref)	0.02
Primary	39	28	23	4.21 (1.40, 12.6)		2.19 (0.62, 5.65)		3.66 (1.35, 9.95)	
None	35	35	34	3.07 (1.05, 8.93)		1.87 (0.62, 5.65)		3.00 (1.12, 8.04)	
Rice straw									
Never used	54	64	59	1 (ref)	0.02	1 (ref)	0.29	1 (ref)	0.04
Used	26	16	21	2.86 (1.10, 7.45)		1.47 (0.71, 3.01)		1.95 (1.03, 3.69)	
Cow dung									
Never used	55	47	43	1 (ref)	0.04	1 (ref)	0.03	1 (ref)	0.009
Used	25	33	37	0.46 (0.21, 1.00)		0.45 (0.21, 0.93)		0.45 (0.24, 0.84)	
Males^#^									
Family h/o cataract									
No	44	59	56	1 (ref)	0.009	1 (ref)	0.08	1 (ref)	0.01
Yes	26	12	16	3.59 (1.27, 10.2)		2.41 (0.86, 6.74)		2.68 (1.22, 5.87)	
Smoking status									
Never smoked	18	28	37	1(ref)	0.12	1(ref)	0.003	1(ref)	0.009
Current or past	52	43	35	2.05 (0.82, 5.11)		3.40 (1.42, 8.14)		2.43 (1.21, 4.88)	
Formal education									
At least secondary	12	27	20	1 (ref)	0.007	1 (ref)	0.39	1 (ref)	0.05
Primary	34	25	31	5.65 (1.67, 19.1)		1.97 (0.62, 6.30)		3.08 (1.18, 8.00)	
None	24	19	21	4.90 (1.23, 19.5)		2.27 (0.67, 7.66)		2.73 (1.00, 7.42)	
Wood or dry leaves									
Never used	47	58	51	1 (ref)	0.01	1 (ref)	0.42	1 (ref)	0.10
Used	23	13	21	3.51 (1.24, 9.95)		1.46 (0.57, 3.74)		1.84 (0.89, 3.83)	

CS = Cases, NE = Non-eye-disease controls; NC = Non-cataract-eye-disease controls.

* Likelihood ratio test.

# Six males who had been exposed to cooking with rice straw and/or cow dung are omitted from this model.

Family history of cataract was strongly and significantly associated with case status in both models within each sex (OR's ranging from 2.14 to 3.59). Lower education was associated with case status in the comparison with NE controls (compared to secondary education or higher, OR's in males and females of 5.65 and 4.21 for primary education, and 4.90 and 3.07 for less than primary). There was also an association with current or past smoking among males in comparison with NC (OR = 3.40).

Overall, associations between use of traditional cooking fuels and case status were more pronounced and significant in the comparison with NE than with NC controls. Nevertheless, as the odds ratios of each fuel in both comparisons were in the same direction, the associations with case status in comparison with the combined control groups are also shown in Table 5. Among females, case status was seen to be positively associated with ever use of rice straw (OR = 1.95, 95%CI 1.03-3.69) but negatively associated with ever use of cow dung (OR = 0.45, 95%CI 0.24-0.84).

The level of exposure to each traditional fuel was expected to be influenced by the level of use of other traditional fuels, and to differ between males and females. For example, lifetime exposure exclusively to wood/dry leaves was lower among exposed males than among females (median 3,650 hours, interquartile range, IQR, 4,732-21,631 hours vs 30,285 hours, IQR 5,096 - 74,256 hours). Parameters reflecting the level of exposure to each fuel were therefore fitted separately to models containing family history of cataract, smoking status and education and using the combined control group. As the different exposure levels in males and females should be reflected in these parameters of the magnitude of exposure, initial models were constructed using all subjects. The patterns of association were similar for each parameter. The model for lifetime exposures, controlling for family history of cataract, education level and smoking status, using all subjects, is shown in upper part of Table 6.

**Table 6:** Conditional logistic regression models for lifetime exposure to cooking fuels.

Cooking fuel	Number of patients		Case vs combined controls		
		
	Case	Control	crude-OR (95% CI)	a-OR (95% CI)	p- value
All subjects					
Wood or dry leaves ever used	47	109	1 (ref)	1 (ref)	0.15
Used ≤25000 hours	55	96	1.67 (0.89, 3.15)	1.93 (0.95, 3.91)	
Used >25000 hours	51	101	1.71 (0.71. 4.11)	2.40 (0.85, 6.79	
Trend				1.52 (0.93, 2.49)	0.10
Rice straw					
Never used	124	266	1 (ref)	1 (ref)	0.07
Used ≤24000 hours	12	16	1.60 (0.73, 3.51)	1.87 (0.74, 4.68)	
Used >24000 hours	17	24	1.61 (0.79, 3.31)	2.18 (0.98, 4.86)	
Trend				1.53 (1.06, 2.24)	0.02
Cow dung					
Never used	127	235	1 (ref)	1 (ref)	0.02
Used≤1500 hours	12	36	0.57 (0.28, 1.17)	0.43 (0.20, 0.92)	
Used >31500 hours	14	35	0.66 (0.33, 1.34)	0.44 (0.20, 0.99)	
Ever used/never used				0.42 (0.23, 0.79)	0.005
Females					
Wood or dry leaves					
Used ≤25000 hours	30	60	1 (ref)	1 (ref)	0.56
Used >25000 hours	50	100	1.00 (0.53. 1.89)	1.27 (0.56, 2.87)	
Rice straw					
Never used	54	123	1 (ref)	1 (ref)	0.09
Used ≤24000 hours	9	13	1.46 (0.61, 3.51)	1.75 (1.62, 5.00)	
Used >24000 hours	17	24	1.61 (0.78, 3.29)	2.21 (0.98, 4.94)	
Trend				1.52 (1.04, 2.22)	0.03
Cow dung					
Never used	55	90	1 (ref)	1 (ref)	0.03
Used≤31500 hours	11	35	0.53 (0.25, 1.11)	0.42 (0.19, 0.93)	
Used >31500 hours	14	35	0.65 (0.32, 1.32)	0.43 (0.22, 0.81)	
Ever used/never used				0.43 (0.22, 0.81)	0.007

* a-OR and likelihood ratio p-values adjusted for family history of cataract and educational level. In addition, smoking status was adjusted for in the all-subjects model.

A significant dose-response relationship with case status was revealed for lifetime exposure to rice straw as a cooking fuel (OR = 1.53, 95%CI 1.06-2.24). However, the odds ratios for lifetime exposure to wood/dry leaves, while suggestive of a trend relationship, were not statistically significant. By contrast, the odds ratios for case status associated with use of cow dung indicated a strong inverse association but with no evidence of a trend relationship (ever vs never use OR = 0.42, 95%CI 0.23-0.79).

In order to confirm these associations, a subsequent model using the subset of females was constructed (Table 6, lower part). This was done to avoid any effect modification due to sex, which might have arisen from the fact that only males were free of any exposure to cooking with traditional fuels and all females had been exposed to cooking with wood and/or dry leaves. Associations between case status and lifetime exposure to cooking with rice straw and with cow dung were almost identical with those seen in the model using all subjects.

Unconditional binary regression models including the same variables as above yielded essentially the same relationships between lifetime exposures and case status as in the conditional models.

## Discussion

This study aimed to test the hypothesis that exposure to cooking with biomass fuels, such as wood or dry leaves, cow dung and rice straw, is significantly associated with the development of cataract among adults less than 50 years of age in rural Bangladesh. After adjusting for family history of cataract, smoking status and level of formal education, differences in exposure to the various biomass fuels were found. Cooking with rice straw was identified as being positively associated with young adult cataract, whereas cooking with cow dung was negatively associated.

The positive association of cooking with rice straw with case status was stronger in comparison with NE than with NC controls. A possible, though unsupported, explanation for this difference depending on the type of controls employed is that the other eye diseases share these risk factors with cataract patients, or that patients with diseases included among the non-eye-disease control group are less exposed to these particular cooking fuels. Inter-comparison of the two types of control, however, revealed no significant associations with the use of these cooking fuels.

It is important to note that, in our study, the association of young adult cataract with using rice straw for cooking among females is relative to the use of other biomass fuels. Because of the sex differences in the patterns of exposure to cooking and the fuels used, the comparator group for males was no exposure to cooking. In contrast, other studies have examined the associations between cataract and the use of cheaper, biomass or solid fuels relative to the use of clean fuels. The use of less expensive cooking fuels was more common among patients with age-related cataract than non-cataract patients in India [6] and, compared with the use of stoves burning clean fuels such as biogas, liquefied petroleum gas or kerosene, the use of solid fuel in unvented stoves was associated with cataract among females of any age in the Nepal-India border area [7]. In both studies the associations remained significant after adjustment for other risk factors, including low educational achievement.

A plausible mechanism underlying the association between the use of certain biomass fuels and development of cataract may be related to the constituents of the large amounts of smoke produced from these fuels damaging the tissues of the eye following either systemic absorption or even local diffusion through the cornea. It has been suggested that such damage may be a result of the endogenous generation of reactive oxygen species by photodynamic action, similar to the purported mechanism by which smoking tobacco may raise the risk of cataract [12,13].

Despite the relationship between risk of cataract and use of cooking fuel being reported in a number of studies, few have attempted to document the associations for different types of biomass fuel. The component materials have either not been specified or have been specified but combined in the analysis and comparisons made with the use of clean fuels. Thus, Mohan (1989) and Mishra (1999) reported elevated risks of both cataract and blindness among an Indian population with exposure to the smoke of biomass cooking fuel, specified as wood, crop residuals and/or cow dung, but separate analyses of each of these materials were not described [14,15].

In view of these reports, the independent inverse association between the use of cow dung as a cooking fuel and case status in our study was unexpected. The relationship held true in comparisons with each type of control. While copious amounts of smoke are known to emanate from burning cow dung, and indoor burning of dung has been reported to produce higher PM_10 _concentrations than that of wood or straw [16], the opposite directions of relationship in our study of rice straw and cow dung might be related to the different complement of smoke constituents. Although cow diet consists largely of fresh grass, bacterial and enzymatic actions of the bovine gastrointestinal tract result in considerable transformations of the plant material.

Constituents of smoke from biomass fuels have been reported to vary considerably with the type of stove employed and with various other differences in the way the fuel is prepared. Comparative information on the constituents of smoke from different biomass fuels, or from dung fuel separate from other biofuels, is scarcely available in the scientific literature, despite several studies of smoke constituents of biomass fuels combined [17-21]. Mudway (2005), however, demonstrated that particles derived from the burning of cow dung cake burned in a traditional Indian cooking stove and deposited in the human respiratory tract lining fluid had considerable oxidative activity, which was mostly due to their transitional metal content [22]. If the postulated mechanism whereby smoke from biomass fuels induces cataract formation through the activity of reactive oxygen species is true, then it is difficult to understand why smoke from cow dung does not have a positive association with young cataract, similar to that of rice straw. Further comparative analyses are required to identify differences in the smoke constituents and elucidate possible differences in the mechanisms of action.

Consideration, however, must also be given to the possibility that the apparent protective effect against the development of young adult cataract of using cow dung as a cooking fuel could be due to uncontrolled confounding. Exposure to cow dung as cooking fuel is more common among middle class families in rural areas in Bangladesh. Cows are usually used for cultivation and dairy products, so more frequently kept by land and farm owners, whereas poor families can rarely afford to buy or keep cattle. Use of cow dung as a fuel thus may be acting as a proxy for higher socioeconomic status, which itself has been identified in some previous studies to be associated with a lower prevalence of cataract (of any type) [1,6,23-25]. Nevertheless, adjusting our models for family income level or for the composite socioeconomic status indicator based on the Kuppuswami scale had no discernable effect on the relationship between case status and use of cow dung as a cooking fuel, so that confounding, if it is to be invoked as the explanation for the relationship, must involve an as yet unidentified variable.

It is of interest, however, that an Indian study of the relationship between fuel use and ocular morbidity in which separate independent associations between different types of cooking fuel and cataract were examined reported a significantly increased risk for wood but not for cattle dung or for gas, kerosene or coal [26]. On the other hand, eye irritation was significantly associated with the use of coal and cattle dung but not the other fuels.

Other variables related to case status in our study - family history of cataract, a history of cigarette smoking, and low educational attainment - have each been recognized as risk factors for cataract in other studies [27-30]. The relationship with low educational achievement may be explained by the generally poorer nutritional status of less educated people. Poor nutritional status [31] as well as experience of dehydrational crises [32], have been identified as independent risk factors for cataract. Unlike the findings of some previous studies [33], working in sunlight was not identified as being associated with case status.

A limitation of this study stems from difficulties in recalling lifetime use of various cooking fuels, although recall was stimulated during the interview by referring to significant life events of each patient. However, it is unlikely that recall misinformation was differential as all patients, both cases and controls, were visiting the hospital for treatment of some ailment, and the specific hypothesis under study was not known to the subjects. Such random errors that may have occurred would therefore tend to reduce the observed strength of association between exposures and outcome. Interviewer knowledge of subject status and the hypothesis under study, which could theoretically introduce bias and an overestimation of associations, is unlikely to have introduced significant distortion of the data as the interviews were carried out strictly according to the structured questionnaire.

Variables of exposure to cooking fuels in this study were confined to those subjects who did the cooking. As other family members could also be exposed, albeit probably to a lower extent, the associations might have been underestimated. Indeed it has been shown that the PM_10 _concentrations in living rooms were only slightly lower than, and closely followed, those in the kitchen throughout the day, in poor households in Bangladesh [16]. Unfortunately, in our study, it is not known whether those subjects who were not exposed to cooking generally remained in the house while cooking was done or were at work away from home.

Both the case-control design of the study and the fact that the relationship between use of rice straw as cooking fuels and development of young adult cataract was not consistently significant in comparisons with the two controls, preclude our drawing firm conclusions regarding a causal relationship between the cooking with rice straw and the risk of developing young adult cataract. The inverse relationship between the use of cow dung and case status, however, was much more consistent. Nevertheless, a plausible explanation for the association is lacking.

The study did not classify the cases with respect to type of cataract. Unless all types share the same risk factors, any heterogeneity of cataract types would have the effect of diluting the true relationships with exposure.

Finally, since this study was conducted in a charitable non-government organization hospital, catering for disadvantaged villagers in remote parts of the country, the range of socio-economic status among subjects was not very wide. Such restricted variability may have prevented the identification of certain risk factors that may be seen in studies with a wider variety of patient backgrounds. On the other hand, the location of the hospital at the time of the study in a poorly accessible rural district with only meagre permanent healthcare facilities makes it unlikely that the cases and controls were drawn from different catchment populations.

The strength of this study lies in its separation of different types of traditional cooking fuel, which allowed the identification of contrasting directions of association among these fuel types.

## Conclusions

Our results may add to the understanding gained from earlier studies regarding the relationship between the use of cheaper biomass cooking fuels and the risk of cataract. Our study provides evidence of differing histories of cooking with several types of biomass fuel between patients developing young adult cataract and control subjects in a population whose cooking fuel exposure is almost exclusively to biomass fuels. Interestingly, the study revealed evidence of lower exposure to cooking with cow dung among cataract patients than among control subjects. In view of the lack of plausible biological explanation for a protective effect of cooking with cow dung and the possibility that the relationship may have resulted from inadequately controlled confounding, further studies of the relationship between exposure to cow-dung smoke and cataract in other settings, as well as comparative analysis of the constituents of the smoke from rice straw and that from cow dung, are needed. Various means of reducing indoor air pollution derived from the use of biomass cooking fuels have been described, such as changing to the use of alternative fuels such as biogas or liquefied petroleum gas, adopting stoves that can reduce the free emission of smoke from biomass fuels, and ensuring adequate ventilation of cooking areas of the house [16,34]. Should the use of rice straw as a cooking fuel be confirmed as a risk factor, then adoption of these pollution-reducing measures may benefit the health of Bangladeshi villagers by reducing the incidence of young adult cataract.

## List of abbreviations used

NE: Non-eye-disease control; NC: Non-cataract eye-disease control; WD/DL: Wood and/or dry leaves; RS: Rice straw; CD: Cow dung; OR: Odds ratio; a-OR: Adjusted odds ratio; CI: Confidence interval.

## Competing interests

The authors declare that they have no competing interests.

## Authors' contributions

JT designed the study, carried out the entire data collection, conducted data analysis and interpretation of the data. AG supervised the entire study and was involved equally during design, data analysis and interpretation of the data. Both authors read and approved the final manuscript

## Pre-publication history

The pre-publication history for this paper can be accessed here:

http://www.biomedcentral.com/1471-2415/11/16/prepub
